# Identification and regulation of expression of a gene encoding a filamentous hemagglutinin-related protein in *Bordetella holmesii*

**DOI:** 10.1186/1471-2180-7-100

**Published:** 2007-11-07

**Authors:** Stefanie Link, Karin Schmitt, Dagmar Beier, Roy Gross

**Affiliations:** 1 Lehrstuhl für Mikrobiologie, Biozentrum der Universität Würzburg, Am Hubland, D-97074 Würzburg, Germany

## Abstract

**Background:**

*Bordetella holmesii *is a human pathogen closely related to *B. pertussis*, the etiological agent of whooping cough. It is able to cause disease in immunocompromised patients, but also whooping cough-like symptoms in otherwise healthy individuals. However, virtually nothing was known so far about the underlying virulence mechanisms and previous attempts to identify virulence factors related to those of *B. pertussis *were not successful.

**Results:**

By use of a PCR approach we were able to identify a *B. holmesii *gene encoding a protein with significant sequence similarities to the filamentous hemagglutinin (FHA) of *B. avium *and to a lesser extent to the FHA proteins of *B. pertussis*, *B. parapertussis*, and *B. bronchiseptica*. For these human and animal pathogens FHA is a crucial virulence factor required for successful colonization of the host. Interestingly, the *B. holmesii *protein shows a relatively high overall sequence similarity with the *B. avium *protein, while sequence conservation with the FHA proteins of the human and mammalian pathogens is quite limited and is most prominent in signal sequences required for their export to the cell surface. In the other Bordetellae expression of the *fhaB *gene encoding FHA was shown to be regulated by the master regulator of virulence, the BvgAS two-component system. Recently, we identified orthologs of BvgAS in *B. holmesii*, and here we show that this system also contributes to regulation of *fhaB *expression in *B. holmesii*. Accordingly, the purified BvgA response regulator of *B. holmesii *was shown to bind specifically in the upstream region of the *fhaB *promoter *in vitro *in a manner similar to that previously described for the BvgA protein of *B. pertussis*. Moreover, by deletion analysis of the *fhaB *promoter region we show that the BvgA binding sites are relevant for *in vivo *transcription from this promoter in *B. holmesii*.

**Conclusion:**

The data reported here show that *B. holmesii *is endowed with a factor highly related to filamentous hemagglutinin (FHA), a prominent virulence factor of the well characterized pathogenic Bordetellae. We show that like in the other Bordetellae the virulence regulatory BvgAS system is also involved in the regulation of *fhaB *expression in *B. holmesii*. Taken together these data indicate that in contrast to previous notions *B. holmesii *may in fact make use of virulence mechanisms related to those described for the other Bordetellae.

## Background

The genus *Bordetella *currently comprises nine species, most of which were found to be associated with host organisms [1,2]. Medically the most important species is *B. pertussis*, the etiological agent of whooping cough for which humans are the only known host. *B. parapertussis *causes milder forms of whooping cough-like disease in humans. *B. bronchiseptica *is known to cause respiratory disease in various mammalian species, but only rarely in humans [2]. These "classical" *Bordetella *species are closely related and the recent determination of their genome sequences confirmed previous suggestions that *B. pertussis *and *B. parapertussis *are independent descendents of *B. bronchiseptica*-like ancestors which during specialization to a single host have sustained a significant erosion of their genetic material [3]. In agreement with their close relationship these organisms produce highly related virulence factors such as several toxins and colonization factors [2].

Among these virulence factors the filamentous hemagglutinin FHA is of particular relevance for pathogenesis. It is an important adhesin and it is required for tracheal colonization in animal models [4]. FHA is a huge protein synthesized as a precursor of 367 kDa which is processed to a mature protein of about 220 kDa by extensive N-terminal and C-terminal modifications involving possibly the Lep signal peptidase and the subtilisin-like autotransporter protein SphB1 [5,6]. FHA is exported to the cell surface and/or secreted via a two-partner export mechanism requiring the FhaC protein located in the outer membrane of the bacteria [7,8]. It has several distinct binding domains involved in adhesion to different substrates. The carbohydrate recognition domain (CRD) probably enables the bacteria to attach to ciliated respiratory epithelial cells and to macrophages [9]. FHA exhibits lectin-like activity for heparin and dextran sulphate possibly involved in the interaction with nonciliated epithelial cells which also contributes to FHA-mediated hemagglutination [10]. Furthermore, there is an Arg-Gly-Asp (RGD) motif which enables FHA to adhere to human monocytes/macrophages via the leukocyte integrin complement receptor 3 (CR3, alpha M beta 2, CD11b/CD18) [11]. The FHA proteins of the "classical" species are highly related, but not entirely functionally exchangeable. Recently, by use of a *B. bronchiseptica *strain expressing the *B. pertussis *FHA protein it was found that the heterologous protein could mediate adherence to various epithelial and macrophage cell lines *in vitro*. In contrast, in rat infection models significant differences in the host-pathogen interaction were noted for the mutant *B. bronchiseptica *strain suggesting significantly different activities of the closely related FHA proteins of the classical species and a role of FHA for host adaptation [12]. Two other proteins, FhaL and FhaS, with significant sequence similarity to FHA are present in the members of the *B. bronchiseptica *cluster, but their functional relevance in virulence is not yet clear [3]. As in case of most other virulence factors, the expression of the *fhaB *locus is controlled by the BvgAS two-component system which is responsive to environmental stimuli such as temperature or compounds such as MgSO_4 _or nicotinic acid [13,14]. The architecture of the *fhaB *promoter and its activation mechanism by the BvgAS system has been investigated in much detail [15,16]. Finally, FHA is a protective antigen and it is included in acellular pertussis vaccines [2].

More recently, additional species were included in the genus *Bordetella*. In 1984, *B avium*, a respiratory pathogen of birds, was first described. The genome sequence of *B. avium *was recently established and revealed the presence of several factors orthologous to those of the "classical" Bordetellae including FHA and the BvgAS two-component system [17,18]. Among other "novel" *Bordetella *species occasionally isolated from patients with underlying disease such as *B. hinzii *and *B. trematum*, *B. holmesii *has gained most attention in the past years, since its 16S rDNA sequence suggested this organism to be most closely related to *B. pertussis *[2,19]. Moreover, *B. holmesii *is known to contain several copies of the insertion elements IS481 and IS1001, otherwise found only in *B. pertussis *and *B. parapertussis*, respectively [20,21]. In addition to its isolation from immunocompromised patients [22-24], *B. holmesii *was found to be able to cause whooping-cough like symptoms in otherwise healthy persons [25,26]. Very little was known about the virulence properties of this bacterium and attempts to identify virulence factors related to those of *B. pertussis *failed. Recently, we succeeded to identify the BvgAS two-component system of *B. holmesii *by PCR amplification with degenerate oligonucleotides [27]. Interestingly, in contrast to the close relationship of the 16S rDNA sequences of *B. holmesii *and *B. pertussis*, the *B. holmesii *BvgAS system was found to be more closely related to the orthologous BvgAS system of *B. avium *than to that of *B. pertussis *[27].

Based again on a PCR approach with degenerate oligonucleotides we attempted to identify additional putative virulence factors of *B. holmesii *related to those of *B. pertussis*. Here we describe the identification and initial characterization of an FHA orthologue of *B. holmesii*. We show that the FHA protein of *B. holmesii *is more closely related to that of *B. avium *than to the FHA proteins of the "classical" Bordetellae. Furthermore, we show that similar to FHA of all other species it is transcriptionally regulated by the BvgAS two-component system suggesting similar virulence strategies in the different *Bordetella *species.

## Results and Discussion

### Identification and sequence analysis of the *fhaB *gene of *B. holmesii*

**Figure 1:**
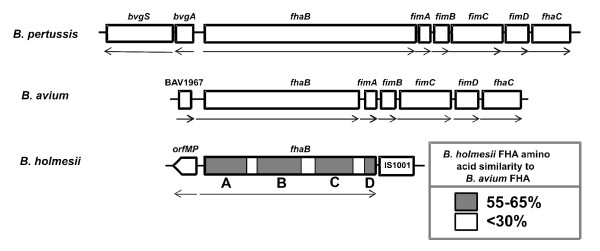
**Schematic view of the gene loci encoding *fhaB*in *B. pertussis*, *B. avium *and *B. holmesii***. Relevant genes are represented as boxes. The arrows below the boxes show the transcriptional polarity of each gene. The open reading frame *orfMP *of *B. holmesii *encoding a putative membrane is not yet completely sequenced. The shaded regions (marked A to D) within the *fhaB *gene of *B. holmesii *indicate the degree of similarity of the deduced protein sequence with that of the FHA protein of *B. avium*. Region A comprises approximately amino acids 1–850, region B amino acids 950 to 1,640, region C amino acids 1,900 to 2,600, and region D amino acids 2,830 to 2,930 of the *B. holmesii *FHA protein; these regions correspond to amino acids 1–820, 840–1,540, 1,600–2310, and 2,520–2621 of the *B. avium *protein, respectively.

Based on short regions of highly conserved nucleotide sequences of the 5'- and 3'-ends of the *fhaB *genes of *B. avium *and *B. pertussis *oligonucleotide pairs Fha1F/Fha1R and Fha2F/Fha2R were designed and used for PCR amplification with chromosomal DNA of *B. holmesii*. For both primer combinations PCR products could be amplified. The sequence analysis of the amplified DNA fragments revealed significant similarity in particular in the deduced amino acid sequence to the respective *fhaB *sequences of the other Bordetellae. By PCR amplification using the primer pair Fha1F/Fha1R, we found that all *B. holmesii *strains tested (*B. holmesii *G7702, *B. holmesii *G8341, *B. holmesii *ATCC51541 and *B. holmesii *No1) harbour orthologues of this *fhaB *gene (data not shown). Using a genome walking strategy the DNA sequence of the *fhaB *gene of strain *B. holmesii *G7702 was completed. The *fhaB *coding region of *B. holmesii *starts with a GTG codon and comprises 8,793 bp with a coding capacity for a 304 kDa protein, which is smaller than FHA of *B. pertussis *(367 kDa) but larger than the respective protein of *B. avium *(273 kDa). The genome organisation of the *fhaB *locus of *B. holmesii *differs from that of the other *Bordetella *species. In *B. holmesii*, upstream of the *fhaB *gene an open reading frame (*orfMP*) is located with significant similarity to a conserved integral membrane protein of *B. bronchiseptica *(BB3004) and *B. parapertussis *(BPP3041) which is transcribed divergently. Directly downstream of the *fhaB *gene a copy of the insertion element IS1001 is located which is known to occur in *B. holmesii *and in *B. parapertussis*. In contrast, in the species belonging to the *B. bronchiseptica *cluster the *fhaB *gene is arranged between the divergently transcribed *bvgAS *locus and the fimbrial operon *fimABCD *which precedes the *fhaC *gene whose gene product is involved in the export of FHA. In *B. avium *an ORF encoding an ABC transporter is present upstream of *fhaB*, while the downstream gene organization is similar to the *B. bronchiseptica *cluster (Fig. 1) [3,17,18].

The overall similarity of the nucleotide sequence of the *fhaB *gene of *B. holmesii *with those of *B. avium *and *B. pertussis *is low (between 50% and 52%) explaining the failure of previous attempts to detect an *fhaB *orthologue in this species by Southern hybridization experiments (data not shown). Even in those regions of the *B. holmesii fhaB *gene (e.g. within the first 600 bp) in which the predicted amino acid sequences of the FHA proteins are quite conserved (see below), the nucleotide sequence similarity is quite limited (59%). On the level of the deduced amino acid sequences, the *B. holmesii *FHA protein shows an overall similarity of 55% to the FHA protein of *B. avium *(Fig. 1). The similarity to the FHA, FhaL and FhaS proteins of the members of the *B. bronchiseptica *complex is less pronounced, e.g. 39.2% to FHA of *B. pertussis *as calculated using the EMBOSS pairwise alignment algorithms  (Table 1). With a sequence similarity of 37.3% the most similar protein outside of the genus *Bordetella *is an adhesin-like protein from *Yersinia frederiksenii*, an environmental bacterium that can also be associated with a wide variety of host organisms [28]. This analysis shows that FHA of *B. holmesii *is more similar to the *B. avium *protein than to the related factors of the mammalian pathogens, in line with previous observations that the BvgA, BvgS and RecA proteins of *B. holmesii *and *B. avium *are more closely related to each other than they are to those of the *B. bronchiseptica *complex [29,30]. This contrasts previous data obtained by comparison of the 16S rDNA sequences, which placed *B. holmesii *as a new species closer to *B. pertussis *than to *B. avium*. On the other hand, recent evidence indicated that the gene encoding the 16S RNA of *B. holmesii *may have been acquired horizontally from *B. pertussis *[30].

**Table 1:** Amino acid sequence similarity of the *B. holmesii *FHA protein and other FHA-like proteins of *Bordetella *species

	**FHA BP**^1)^	**FhaL BP**	**FhaS BP**	**FHA BB**	**FHA BA**
**FHA BH**	39.2/28.1^2)^	29.4/19.9	37.3/26.1	38.0/26.9	55.5/41.5
**FHA BP**		31.5/22.7	49.2/38.8	91.2/89.0	40.0/27.7
**FhaL BP**			30.7/23.1	29.6/21.1	29.2/19.4
**FhaS BP**				43.3/37.3	41.3/27.5
**FHA BB**					39.2/26.8

^1) ^BP: *B. pertussis*; BB: *B. bronchiseptica*; BA: *B. avium*; BH: *B. holmesii*

^2) ^Sequence similarity in %/Sequence identity in %

For the Sec-dependent secretion across the cytoplasmic membrane, the *B. holmesii *FHA protein has an extended N-terminal signal sequence, which is very similar to that of the *B. pertussis *protein. In *B. pertussis*, the signal sequence is cleaved at an alanine at sequence position 71 probably by the Lep signal peptidase [6]. The *B. holmesii *FHA protein has also an alanine residue at this position suggesting its processing at this site after transport through the cytoplasmic membrane. In addition, the N-terminus of *B. pertussis *FHA harbours a domain about 250 amino acid residues in length which is essential for secretion through the outer membrane according to the two-partner secretion model, the so-called TPS domain [31]. The exact nature of the transport signal is not known so far, but two sequence motifs (NPNL and NPNGI) are well conserved among two-partner secreted proteins and at least the NPNL motif plays a role in the secretion of *B. pertussis *FHA [32]. Both sequence motifs are also present in the FHA protein of *B. holmesii *suggesting that it is also exported via a two-partner secretion mechanism.

**Figure 2:**
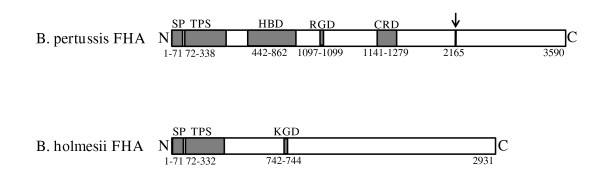
**Schematic representation of the overall structureof FHA proteins of *B. pertussis *and *B. holmesii***. Protein regions with known or presumable functional importance are represented by dark grey boxes. The arrow indicates the maturation site for proteolytic cleavage of the *B. pertussis *FHA by the SphB1 protease. Abbreviations: N, N terminus; C, C terminus; SP, signal peptide; TPS, two-partner secretion domain; HBD, heparin binding domain; RGD, arginine-glycine-aspartic acid motif; CRD, carbohydrate binding motif; KGD, lysine-glycine-aspartic acid motif. Numbers indicate amino acid positions within the FHA proteins.

Similarities of the *B. holmesii *protein with domains of the *B. pertussis *FHA protein possibly involved in adhesion including the heparin binding (aa 442–862) and carbohydrate recognition domains (CRD) (aa 1141–1279) are very limited and the elucidation of relevant binding activities must await the biochemical characterization of the *B. holmesii *FHA protein. An intriguing feature of the FHA protein of *B. pertussis *is the presence of an RGD motif (aa 1097–1099) enabling the protein to interact with integrin receptors [2]. The *B. holmesii *protein does not contain an RGD motif, instead at sequence position 742–744 it harbours a KGD motif (Fig. 2). In some cases, KGD motifs may be involved in integrin binding [33,34], however, ascribing such a function to the KGD motif of the *B. holmesii *protein must await future experimental analysis.

### Regulation of expression of the *B. holmesii fhaB *gene

**Figure 3:**
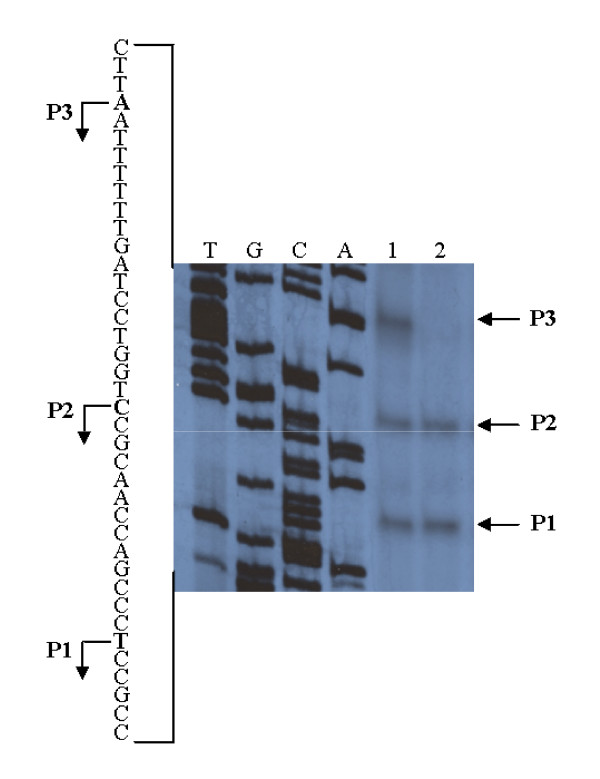
**Determination of transcriptional start sites of the *gfp *reporter gene fused to the upstream region of *fhaB *of *B. holmesii *by primer extension analysis**. Equal amounts of total RNA extracted from *B. holmesii *G7702 (pMMB208-*fhaP-gfp0*) (lane 1) and *B. holmesii *G7702 *bvgA *(pMMB208-*fhaP-gfp0*) (lane 2) were hybridized with radiolabelled oligonucleotide gfp.PE. The cDNAs corresponding to the transcripts P1 to P3 are indicated by arrows. A part of the *fhaB *promoter sequence is shown on the left. Transcriptional start points are indicated by arrows. The sequencing reaction (lanes A, C, G and T) was performed using oligonucleotide gfp.PE and plasmid pSK-*fhaP-gfp0 *as a template.

Upstream of the *fhaB *gene a divergently transcribed gene is present suggesting that the *fhaB *gene has a promoter of its own. To analyse the regulatory region of the *fhaB *gene (P_*fhaB*_) it was cloned upstream of a promoterless *gfp *gene in the broad host range vector pMMB208 and the resulting plasmid was introduced into *B. holmesii*. By primer extension analysis with a *gfp*-specific primer three transcripts (P1 – P3) were identified initiating at sequence positions -58 (P1), -71 (P2) and -88 (P3) with respect to the translational start codon of the *fhaB *gene (Fig. 3). No obvious -10 and -35 regions could be observed upstream of any of these putative transcriptional start sites. To investigate whether, similar to the other Bordetellae, the BvgAS system is involved in the control of *fhaB *expression, primer extension analysis was performed on RNA extracted from a *B. holmesii bvgAS *mutant [27] harbouring the plasmid-borne P_*fhaB*_-*gfp *fusion. Interestingly, the longest of the three transcripts (P3) was not detected in the *bvgAS *mutant indicating that the synthesis of this transcript is controlled by the BvgAS system, while the other transcripts are constitutively produced under our experimental conditions (Fig. 3). Similarly, the *fhaB *gene of *B. pertussis *is controlled by a BvgAS regulated promoter, but some constitutive expression was noted [35].

Although the *fhaB *gene is transcribed in *B. holmesii *grown at standard culture conditions, various attempts to detect the *B. holmesii *FHA protein were not successful so far. The *B. holmesii *strains used in this study grow very poorly in liquid culture reaching a maximal OD_600 _of about 0,5. We were unable to detect a protein with a molecular weight corresponding to FHA in the culture supernatants neither by SDS PAGE nor by immune blotting using a polyclonal antiserum against the *B. pertussis *FHA protein. Similarly, also in whole cell lysates of bacteria grown on BG agar plates no FHA protein could be detected (data not shown). It is therefore possible, that the translation efficiency of the *fhaB *gene is low, which may be in line with the fact that the open reading frame starts with a GTG codon, or that the protein is processed to smaller fragments than the related proteins of the other Bordetellae.

**Figure 4:**
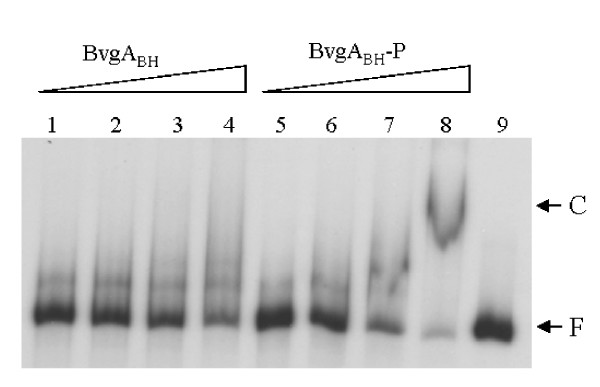
**Binding of BvgA_BH _to the *fhaB *upstream region of *B. holmesii***. A radiolabelled 277 bp PCR fragment containing the *fhaB *upstream region of *B. holmesii *was incubated with 150, 350, 500 and 650 ng of unphosphorylated (lanes 1–4) and *in vitro *phosphorylated (lanes 5–8) BvgA_BH_, respectively. Lane 9 contains the radiolabelled DNA probe. The reaction mixtures were run on a non-denaturating 4% polyacrylamide gel. F, free DNA; C, DNA-protein complex.

**Figure 5:**
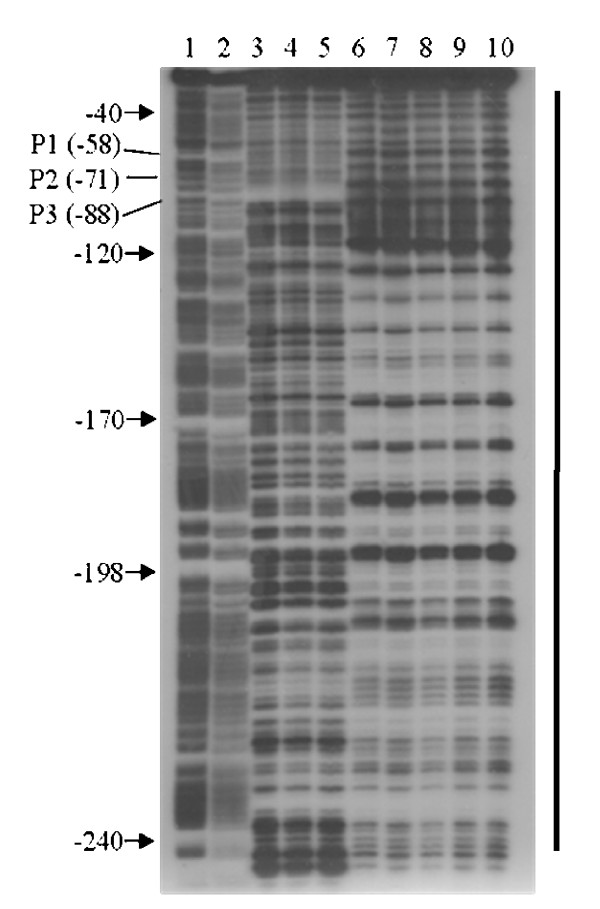
**Binding of the *B. holmesii *BvgA_BH _protein to the *fhaB *upstream region of *B. holmesii***. The footprint shows the entire region protected by BvgA_BH _as indicated by the bar on the right side of the figure. DNase I footprint experiments were performed on a 312 bp BamHI-HindIII DNA fragment from plasmid pSK-FP labelled at its BamHI site containing the entire *fhaB *upstream sequence including all four putative binding sites (BS1–BS4). The 5'-labelled probe was incubated with 0.5, 1.0, 2.0, 3.0, 4.0, 6.0 and 8.0 μg of *in vitro *phosphorylated BvgA_BH _(lanes 4–10, respectively). No protein was added to the reaction mixture loaded in lane 3. Lane 1 and 2 are G+A sequencing reactions on the DNA probe used as a size marker [42]. Numbers on the left indicate the distance from the translational start codon of the *fhaB *gene. The positions of the start sites of transcripts P1–P3 are indicated.

To further investigate the transcriptional regulation of the *fhaB *gene by the BvgAS system we performed DNA binding experiments *in vitro *with purified recombinant BvgA_BH _of *B. holmesii *[27]. In fact, in band shift experiments binding of the phosphorylated but not of the unphosphorylated BvgA_BH _protein to the *fhaB *upstream region could be detected (Fig. 4). Binding was specific since addition of unspecific competitor DNA did not interfere with binding of BvgA_BH_-P even in the presence of a 1,000 fold excess of competitor DNA (data not shown). To further characterize BvgA binding to the promoter region, DNase I footprint analysis with BvgA_BH _of *B. holmesii *was performed. Footprint experiments were carried out on a 312 bp DNA segment ranging from nucleotide position +29 to -283 as numbered with respect to the translational start site of the *fhaB *gene. The addition of BvgA_BH_-P to the reaction mixture resulted in a large region protected from DNase I digestion ranging from position -40 to -243 with respect to the start codon of *fhaB*. Within the protected region the appearance of a regular pattern of hypersensitive sites every 10 to 11 nucleotides could be observed (Fig. 5), a phenomenon which was noted previously in the case of the promoter of the *bvgAS *operon of *B. holmesii *[27]. Surprisingly, the protected area covers all three transcriptional start sites mapped by primer extension analysis and, accordingly, includes the corresponding core promoter elements. It is not clear whether this observation has *in vivo *relevance.

**Figure 6:**
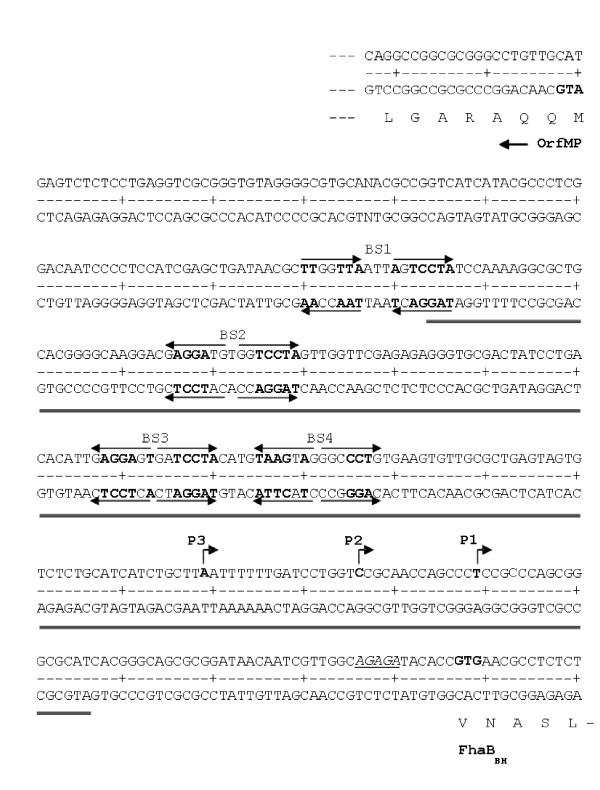
**Representation of the intergenic region between *orfMP *and *fhaB *of *B. holmesii***. Partial amino acid sequences are shown below the respective coding DNA sequences. The GTG start codon of *fhaB *and the ATG start codon of the neighbouring *orfMP *coding for a putative membrane protein are given in bold letters. The putative Shine/Dalgarno sequence of *fhaB *is shown in italics and underlined. Transcriptional start sites of the constitutively synthesized transcripts P1 and P2 and of the *bvg*-dependent transcript P3 of *fhaB *are marked by arrows. The region protected from DNaseI digestion in footprint experiments with BvgA_BH_-P is underlined. The sequence motifs BS1 to BS4 showing similarity to the BvgA consensus binding site in *B. pertussis *promoters are indicated by horizontal arrows above and below the DNA sequence. Nucleotides which match the consensus sequence are marked in bold letters.

A search for putative BvgA binding sites within the *fhaB *promoter region revealed the presence of four sequence motifs termed BS1 to BS4, respectively, with similarities to the well defined BvgA binding sites in *B. pertussis *(Fig. 6) which are located within or close to the region protected by BvgA_BH_-P in the footprint experiments. BS2 and BS3 show high similarity to each other and consist of inverted repeat heptanucleotide sequences centered at position -117 and -65.5 relative to the start site of transcript P3. The left and right half-site motifs of BS2 and BS3 match the consensus half-site motif for binding of *B. pertussis *BvgA (BvgA_BP_) [15] in 4 and 5 (BS2) and 5 (BS3) out of seven positions. BS1 is arranged as a direct heptanucleotide repeat whose half-sites match the consensus in 5 and 6 positions, respectively, and is centered at position -161. BS4 which consists of an inverted repeat centered at position -47.5 shows the lowest similarity to the consensus heptanucleotide BvgA_BP _binding motif (4 and 3 matches per half-site). The *fhaB *promoter of *B. pertussis *comprises a heptanucleotide inverted repeat sequence with high affinity for BvgA_BP _binding centered at position -88.5 relative to the transcriptional start, as well as two additional low affinity binding sites centered at position -67.5 and directly adjacent to the -35 region [14,15]. Cooperative binding of BvgA_BP _to the secondary binding sites which show only limited similarity to the high-affinity inverted repeat motif is required for full transcriptional activation of the *fhaB *promoter of *B. pertussis *[15,35]. Remarkably, the positions of the low-affinity BvgA_BP _binding sites in the *fhaB *promoter of *B. pertussis *and of BS3 and BS4 in the upstream region of *fhaB *from *B. holmesii *are almost identical. However, while the high-affinity binding site in the *fhaB *promoter of *B. pertussis *is located immediately 5' adjacent to the low-affinity sites, the centers of the inverted repeat sequences BS2 and BS3 are located in a distance of 51 bp. The most prominent sites showing hypersensitivity to DNase I cleavage in footprint experiments map to the region flanked by BS2 and BS3 (Fig. 5).

**Figure 7:**
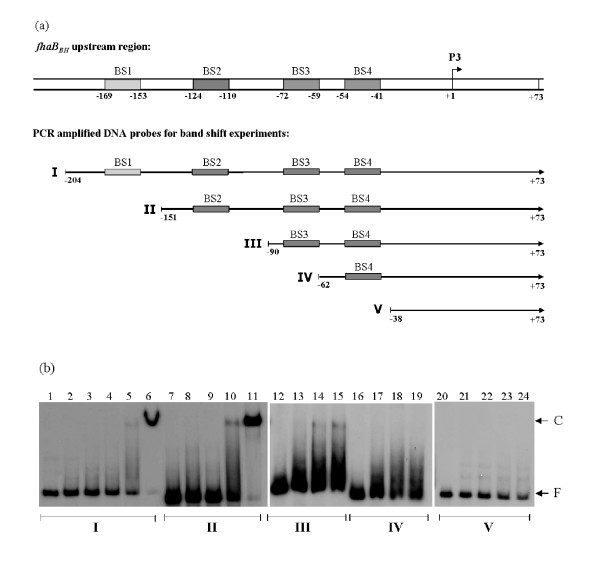
**Characterization of putative BvgA binding sitesinside the *fhaB *upstream region of *B. holmesii***. (a) Schematic representation of the *fhaB *upstream region containing the four putative BvgA binding sites BS1–BS4 and representation of the PCR amplified DNA probes I-V used for band shift experiments with the purified *B. holmesii *BvgA_BH _protein. Numbers indicate the distance from the Bvg-dependent *fhaB *transcriptional start site (P3) taken as position +1. (b) Binding of the *B. holmesii *BvgA_BH _protein to DNA probes I-V, containing different amounts of putative BvgA binding sites (a); the radiolabelled PCR fragments were incubated with 150 ng (lane 2), 300 ng (lanes 3 and 8), 400 ng (lanes 4 and 9), 500 ng (lanes 5, 10, 13, 17 and 21), 600 ng (lane 22), 700 ng (lanes 6, 11, 14, 18 and 23) and 800 ng (lanes 15, 19 and 24) of *in vitro *phosphorylated BvgA_BH_. No protein was added in lane 1 (DNA probe I), lane 7 (DNA probe II), lane 12 (DNA probe III), lane 16 (DNA probe IV) and lane 20 (DNA probe V). The reaction mixtures were run on a non-denaturating 4% polyacrylamide gel. F, free DNA probes; C, DNA-protein complexes.

To investigate a functional role of these putative BvgA binding site(s) we performed a deletion analysis of the *fhaB *promoter region and carried out band shift assays with progressively 5'- truncated DNA fragments lacking BS1 to BS4. As shown in Fig. 7, BvgA_BH_-P bound equally well to DNA fragments comprising the four putative BvgA binding sites BS1 to BS4 and to a DNA fragment lacking BS1 suggesting a negligible role of BS1 for the activation of the BvgA-P dependent promoter of *fhaB*. In agreement with this assumption, BS1 was only partially protected by BvgA_BH_-P binding in DNase I footprint experiments (Fig. 6). When BvgA_BH_-P was incubated with a DNA fragment lacking both BS1 and BS2 still a distinct DNA-protein complex was formed, however, binding was significantly weaker since a much higher amount of BvgA_BH_-P was required to achieve a band shift. No significant binding of BvgA_BH_-P was detectable to DNA fragments containing only BS4 or no BS box at all. These data suggest a functional role of the highly similar BS2 and BS3 sites for binding of BvgA_BH_-P to the *fhaB *upstream region.

**Figure 8:**
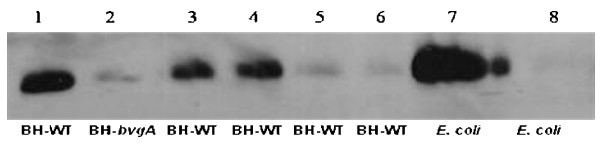
**Immunoblot analysis of protein lysates of *B. holmesii *strains with a polyclonal anti-GFP antiserum**. *B. holmesii *G7702 (pMMB208-*fhaP-gfp0*; BS1 to BS4) (lane 1), *B. holmesii *G7702 *bvgA *(pMMB208-*fhaP-gfp0*; BS1 to BS4) (lane 2), *B. holmesii *G7702 (pMMB208-*fhaP-gfp2*; BS2 to BS4) (lane 3), *B. holmesii *G7702 (pMMB208-*fhaP-gfp3*; BS3 and BS4) (lane 4), *B. holmesii *G7702 (pMMB208-*fhaP-gfp4*; BS4) (lane 5), *B. holmesii *G7702 (pMMB208-*fhaP-gfp6*; no BS) (lane 6). Lysates of *E. coli *(pSK-*fhaP-gfp0*) expressing GFP under control of the pSK *lac *promoter (lane 7) and of *E. coli *(pMMB208-*fhaP-gfp0*) (lane 9) were analysed as positive and negative control, respectively. BH-WT, *B. holmesii *wild type; BH-*bvgA*, *B. holmesii bvgA *mutant.

To test whether these *in vitro *data have also relevance *in vivo*, DNA fragments containing various pieces of the *fhaB *upstream region were cloned in a promoterless *gfp *expression vector and transferred to the *B. holmesii *wild type and the *bvgAS *mutant strain. GFP expression directed from the different constructs was used as a measure for promoter activity. GFP expression was strong in the *B. holmesii *wild type strain harbouring construct pMMB208-*fhaP-gfp0 *containing the entire *fhaB *upstream region, while very low amounts of GFP were detected in the *bvgAS *mutant harbouring the same plasmid (Fig. 8, compare lanes 1 and 2). These data confirm the BvgAS mediated regulation of *fhaB *expression which was already observed on the transcriptional level (Fig. 3). Moreover, corroborating the *in vitro *data, GFP expression was virtually absent in strains *B. holmesii *G7702 (pMMB208-*fhaP-gfp4*) and *B. holmesii *G7702 (pMMB208-*fhaP-gfp6*) whose *gfp *expression plasmids contained only site BS4 or did not contain a BS site at all (Fig. 8, lanes 5 and 6). In agreement with the results of the DNA binding experiments a dramatic increase in GFP expression could be noted when the *fhaB *upstream region in the *gfp *expression plasmids comprised BS3 or BS2 and BS3 in addition to BS4 (Fig. 8, lanes 4 and 3). The virtually identical GFP expression directed from the pMMB208-*fhaP-gfp3 *(containing BS3 and BS4) and pMMB208-*fhaP-gfp2 *(containing BS2 to BS4) plasmids was surprising since the EMSA studies reported above revealed a relatively weak binding of BvgA_BH_-P to a promoter fragment containing BS3 and BS4, while binding to a fragment comprising BS2 to BS4 was very efficient suggesting a prominent role of BS2 for transcriptional activation. Since BS3 is fairly similar to the consensus BvgA_BP _binding motif and is located at the appropriate position to allow the interaction between BvgA_BH _and the C-terminal domain of the α subunit of RNA polymerase, BvgA_BH _binding to BS3 facilitated by DNA topology effects might be sufficient to fully activate the plasmid-borne *fhaB *promoter in pMMB208-*fhaP-gfp3*. In the presence of the full length *fhaB *promoter cooperative protein interactions are likely be involved in the binding of BvgA_BH _to the BS2/BS3 region.

To investigate whether the *fhaB *promoter of *B. holmesii *is also recognized by the BvgA protein of *B. pertussis*, the pMMB208-*fhaP-gfp0 *plasmid containing the entire promoter region of the *B. holmesii fhaB *gene fused to GFP was introduced into the *B. pertussis *strains Tohama I (TI) and BP359. Strong GFP expression was observed by immunoblot analysis in the wildtype strain TI (pMMB208-*fhaP-gfp0*), while expression of the reporter gene was hardly detectable in the *bvgAS *mutant BP359 (pMMB208-*fhaP-gfp0*) (data not shown). Interestingly, primer extension analysis performed with RNA extracted from TI (pMMB208-*fhaP-gfp0*) and BP359 (pMMB208-*fhaP-gfp0*) using a *gfp*-specific oligonucleotide demonstrated that in the wild type strain transcription of *gfp *starts at two sites, which, however, are identical to the start sites of transcripts P1 and P3 synthesized from constitutive (P1) and *bvg*-dependent (P3) dependent promoters in *B. holmesii*. Moreover, as observed in *B. holmesii*, in *B. pertussis *the promoter directing the synthesis of transcript P3 is not transcribed anymore when the BvgAS system is inactivated (data not shown). These data suggest that the activation mechanism of the *fhaB *promoter of *B. holmesii *by the BvgA proteins of *B. holmesii *and *B. pertussis *is remarkably similar. This is surprising since it was recently shown that the BvgA protein of *B. holmesii *does not bind to and cannot activate the *fhaB *promoter of *B. pertussis*, although in particular in its C-terminal output domain it is highly related to the BvgA protein of *B. pertussis *[27,36].

## Conclusion

Little was known so far about the virulence mechanisms of *B. holmesii *which can cause pertussis-like disease in humans and within the genus *Bordetella *was thought to be most closely related to *B. pertussis*. Previous attempts to identify possible virulence factors related to those of the etiological agent of whooping cough and of the other well-characterized Bordetellae were not successful. Here we describe the identification of a *B. holmesii *factor related to the major adhesin of the other pathogenic Bordetellae, the filamentous hemagglutinin FHA. This adds to our previous report on the identification of a two-component system in *B. holmesii *orthologous to the BvgAS two-component system of *B. pertussis *which in the other pathogenic Bordetellae is the master regulator of virulence gene expression and directly controls the expression of FHA. We show that also in *B. holmesii *the expression of FHA is regulated by the BvgAS system and that the activation mechanism of the *fhaB *promoter in *B. holmesii *resembles that in *B. pertussis*. These data strongly suggest that basic virulence mechanisms of *B. holmesii *and of the other pathogenic Bordetellae are related. Furthermore the present study provides further evidence that *B. holmesii *may be more closely related to the bird pathogen *B. avium *than to *B. pertussis *indicating that in the genus *Bordetella *in different phylogenetic lineages independent strains repeatedly evolved towards being human pathogens.

## Methods

### Bacterial strains and growth conditions

Bacterial strains used in this study are listed in Table 2. *B. holmesii *strains, *B. pertussis *strains and *B. bronchiseptica *strains were grown on Bordet-Gengou (BG) agar plates supplemented with 20% horse blood [27]. When required, antibiotics were added to the following final concentrations: streptomycin, 100 μg ml^-1^; spectinomycin, 100 μg ml^-1^; kanamycin, 50 μg ml^-1^; gentamycin, 15 μg ml^-1^; ampicillin, 100 μg ml^-1 ^and chloramphenicol, 20 μg ml^-1^. Bacterial conjugations were performed as described previously [37], using *Escherichia coli *SM10 as the donor strain [38]. Protein lysates were prepared from bacteria grown on BG agar plates for 48 h at 37°C which were suspended in saline at a cell density of 1.4 × 10^8 ^c.f.u. ml^-1^.

**Table 2:** Bacterial strains and plasmids

**Bacterial strain or plasmid**	**Relevant feature(s)**	**Reference or source**
**Strains**		
*B. holmesii*		
*ATCC 515*41	clinical isolate	[19]
No1	clinical isolate	[23]
G8341	clinical isolate	[19]
G7702	clinical isolate	[19]
G7702 *bvgA*	G7702 with a kanamycin-resistance cassette disrupting the *bvgA *gene	[27]
*B. pertussis*		
Tohama I (TI)	wildtype, but *rpsL*	[43]
BP359	derivative of TI, *bvgA*::Tn5	[43]
*E. coli*		
DH5α	strain used for high-efficiency transformation	Gibco
SM10	mobilizing strain	[38]
**Plasmids**		
PbluescriptSK	high copy number cloning vector	Stratagene
pMMB208	Broad-host-range expression vector	[44]
pKEN2	plasmid containing the promoterless *gfp*-mut2 gene	[40]
pSK-FP	pSK carrying a 312 bp PCR fragment of *B. holmesii *G7702 derived from the upstream region of *fhaB*	This study
pSK-*fhaP-gfp0*	pSK carrying a fusion between 277 bp of the *fhaB *promoter region of *B. holmesii *G7702 harbouring the putative bindings sites BS1–BS4 and the *gfp*-mut2 gene	This study
pMMB208-*fhaP-gfp0*	pMMB208 carrying a fusion between 277 bp of the *fhaB *promoter region of *B. holmesii *G7702 (BS1–BS4) and the *gfp*-mut2 gene	This study
pMMB208-*fhaP-gfp2*	pMMB208 carrying a fusion between 224 bp of the *fhaB *promoter region of *B. holmesii *G7702 (BS2–BS4) and the *gfp*-mut2 gene	This study
pMMB208-*fhaP-gfp3*	pMMB208 carrying a fusion between 168 bp of the *fhaB *promoter region of *B. holmesii *G7702 (BS3–BS4) and the *gfp*-mut2 gene	This study
pMMB208-*fhaP-gfp4*	pMMB208 carrying a fusion between 146 bp of the *fhaB *promoter region of *B. holmesii *G7702 (BS4) and the *gfp*-mut2 gene	This study
pMMB208-*fhaP-gfp6*	pMMB208 carrying a fusion between 111 bp of the *fhaB *promoter region of *B. holmesii *G7702 and the *gfp*-mut2 gene	This study

### General techniques

DNA manipulation, cloning procedures and acrylamide gel electrophoresis were carried out according to standard procedures. PCR amplifications were performed with a model T3 thermocycler (Biometra) using Pfu polymerase (Promega) or Taq polymerase (Qbiogene Inc.). Oligonucleotides used in this study are listed in Table 3. All cloned PCR products were subjected to automated sequencing to ensure proper amplification. Immunoblot analysis was performed using a semidry blotting procedure as described previously [39]. Green fluorescent protein (GFP) was detected using rabbit GFP antiserum (Invitrogen).

**Table 3:** Oligonucleotides used in this study

**Oligonucleotide**	Sequence (5'-3')*	**Restriction sites**
Fha1F	5'-CTCATCATCGCCAACCCCAACGG -3'	-
Fha1R	5'-AGCTGGCGCACGCCCAGGCCTG -3'	-
Fha2F	5'-CCCAAGCCCAAGCCCAAGCCCAAGGCC -3'	-
Fha2R	5'-ATAGAAGACCCGGTAGTTCT -3'	-
FhaBamHI	5'-CCTCGGAGGATCCCCTCCATCGA -3'	BamHI
FhaHindIII	5'-TACTTTGCTGAAGCTTAAACGATAG -3'	HindIII
Fhagfp1	5'-CCTCGGAGGATCCCCTCCATCGA -3'	BamHI
Fhagfp2	5'-ACAACGAGAGGATCCGCAGCAA -3'	BamHI
Fhagfp3	5'-CAAAAGGGGATCCACGGGGCAA -3'	BamHI
Fhagfp4	5'-AGGGTGCGAGGATCCTGACACA -3'	BamHI
Fhagfp6	5'-AAGTGTTGGGATCCGTAGTGTCT -3'	BamHI
Fhagfp7	5'-AACGATCTAGATCCGCGCTGCCC -3'	XbaI
FhaGR1	5'-GACTATCCTGACACATTGAGGAG -3'	-
FhaGR2	5'-CTACATGTAAGTAGGGCCCTGTG -3'	-
Gfp1	5'-CAAGAATTGGGACAACTCCAGT -3'	-

* Restriction sites introduced for cloning purposes are underlined.

### Characterization of the *fhaB *locus of *B. holmesii*

Chromosomal DNA of *B. holmesii *G7702 was used as template for PCR reactions. Primers for PCR reactions were deduced from conserved DNA regions of the *fhaB *gene of *B. pertussis *and *B. avium*. Primer pair Fha1F/Fha1R was deduced from the 5'-end of the *fhaB *gene (Fha1F: base pair 514 to 536 in *fhaB *of *B. pertussis*; base pair 493 to 515 in *fhaB *of *B. avium*; Fha1R: base pair 923 to 944 in *fhaB *of *B. pertussis*; base pair 902 to 923 in *fhaB *of *B. avium)*. Primer pair Fha2F/Fha2R was deduced from the 3'-end of the *fhaB *gene (Fha2F: base pair 10420 to 10445 in *fhaB *of *B. pertussis*; base pair 7573 to 7598 in *fhaB *of *B. avium*; Fha2R: base pair 10738 to 10757 in *fhaB *of *B. pertussis*; base pair 7877 to 7896 in *fhaB *of *B. avium*). Using primer pairs Fha1F/Fha1R and Fha2F/Fha2R, two fragments of the expected length (340 bp and 440 bp) could be amplified from chromosomal DNA of *B. holmesii *G7702. The PCR products were sequenced and the sequence analysis demonstrated that the DNA fragments encoded part of the *fhaB *homologue of *B. holmesii*. The entire *fhaB *gene of *B. holmesii *was sequenced by a genome walking approach using the Universal Genome Walker Kit (Clontech Inc.).

### Construction of *B. holmesii *and *B. pertussis *strains containing a plasmid with a fusion of the *fhaB *promoter region of *B. holmesii *to a *gfp *reporter gene

A 277 bp DNA fragment containing the entire promoter region of the *fhaB *gene was PCR amplified from genomic DNA of *B. holmesii *G7702 using the primer pair Fhagfp1/Fhagfp7, thereby introducing BamHI and XbaI restriction sites at the 5'- and 3'-terminus, respectively. A DNA fragment containing the promoterless *gfp*-mut2 gene was excised with XbaI and HindIII from plasmid pKEN [40]. The 277 bp DNA fragment harbouring the *fhaB *promoter (termed *fhaP0*) and the *gfp *fragment were cloned together in plasmid pSK, resulting in plasmid pSK-*fhaP-gfp0*. The *fhaP-gfp0 *fragment was then excised by BamHI- and HindIII-digestion and was subsequently ligated into plasmid pMMB208. In the resulting plasmid pMMB208-*fhaP-gfp0*, the fusion of the promoter fragment and the *gfp *gene is located in the opposite orientation to the plasmid-borne *tac *promoter. pMMB208-*fhaP-gfp0 *was subsequently transformed into *E. coli *SM10 and transferred by conjugation into various *B. holmesii *and *B. pertussis *strains. The same protocol was applied to generate the following constructs, which contain fusions of different *fhaB *promoter fragments of *B. holmesii *G7702 with the *gfp *reporter gene: pMMB208-*fhaP-gfp2 *(*fhaP2*, 224 bp, amplified with Fhagfp2/Fhagfp7), pMMB208-*fhaP-gfp3 *(*fhaP3*, 168 bp, amplified with Fhagfp3/Fhagfp7), pMMB208-*fhaP-gfp4 *(*fhaP4*, 146 bp, amplified with Fhagfp4/Fhagfp7), and pMMB208-*fhaP-gfp6 *(*fhaP6*, 111 bp, amplified with Fhagfp6/Fhagfp7).

### Primer extension experiments

Total RNA was prepared from bacteria grown on BG agar plates for 48 h at 37°C. Primer extension experiments were carried out essentially as described previously [27] with the primer oligonucleotide Gfp1 (Table 2). Sequencing reaction mixtures, with plasmid pSK-*fhaP-gfp0 *as template DNA and the appropriate oligonucleotide primer, were analysed on 6% urea-polyacrylamide gels and used as standards for determination of the transcription initiation sites.

### Gel retardation experiments

A 277 bp DNA fragment (probe I) containing part of the *fhaB *upstream region was PCR amplified from genomic DNA of *B. holmesii *G7702 using primer pair Fhagfp1/Fhagfp7. The PCR fragment was 5'-end labelled with [γ-^32^P]-ATP using T4 polynucleotide kinase (MBI) and purified using the QIAquick Nucleotide Removal Kit (Qiagen Inc.). The His_6_-BvgA_BH _protein described previously [27] was diluted in 1 × dilution buffer (2 mM MgCl_2_, 50 mM KCl, 0.1% Igepal CA 630, 10 mM DTT) and was phosphorylated by incubation with 50 mM acetyl phosphate (Sigma Inc.) for 20 min at room temperature. Increasing amounts of the protein were added to approximately 15,000 cpm of the labelled DNA probe in 20 μl of 1 × binding buffer (10 mM Tris/HCl, pH 8, 10 mM KCl, 5 mM EDTA, 1 mM DTT, 10% glycerol, v/v). The samples were incubated for 20 min at room temperature and were then loaded onto a non-denaturing 4% polyacrylamide gel. Gels were run for 2.5 h at 150 V and subsequently the dried gels were autoradiographed. The same procedure was applied using the following DNA probes, which were amplified from the *fhaB *upstream region of *B. holmesii *G7702: probe II (224 bp, amplified by Fhagfp2/Fhagfp7), probe III (163 bp, amplified by FhaGR1/Fhagfp7), probe IV (135 bp, amplified by FhaGR2/Fhagfp7) and probe V (111 bp, amplified by Fhagfp6/Fhagfp7).

### DNase I footprinting

DNase I footprint experiments were performed essentially as described previously [41]. A 312 bp DNA fragment containing part of the upstream region of the *fhaB *gene was PCR amplified from chromosomal DNA of *B. holmesii *G7702 using primer pair FhaBamHI/FhaHindIII, thereby introducing BamHI and HindIII restriction sites at the 5'- and 3'-terminus, respectively. The purified *fhaB *upstream fragment was cloned into plasmid pSK. The resulting plasmid pSK-FP was digested with BamHI and 5'-end labelled with [γ-^32^P]-ATP using T4 polynucleotide kinase. The labelled promoter fragment was excised from the plasmid by HindIII digestion, purified by gel electrophoresis and eluted in 4 ml elution buffer (10 mM Tris/HCl, pH 8, 1 mM EDTA, 300 mM sodium acetate, 0.2% SDS). The eluted probe was then extracted with phenol/chloroform (1:1, v/v) and ethanol precipitated. Binding reaction mixtures contained various concentrations of BvgA_BH _protein and approximately 100,000 cpm of labelled DNA probe in 50 μl of 1 × binding buffer (10 mM Tris/HCl, pH 8, 2 mM MgCl_2_, 0.1 mM CaCl_2_, 1 mM DTT, 10% glycerol, v/v). The samples were incubated 20 min at room temperature and then the nucleolytic reactions were initiated by the addition of 1 U DNase I in 1 × binding buffer. After 1 min digestions were terminated by the addition of 140 μl stop buffer (192 mM sodium acetate, 0.14% SDS, 62 μg ml^-1 ^yeast tRNA). The samples were extracted with phenol/chloroform (1:1, v/v), ethanol precipitated and run on a 6% polyacrylamide-urea sequencing gel. A G+A sequencing reaction was also conducted in parallel with the labelled DNA probe and subjected to electrophoresis on the same gel [42].

### Accession Number

The DNA sequence reported in this manuscript can be retrieved by the accession number [EMBL:AM491633].

## Authors' contributions

SL: Carried out molecular genetic experiments

KS: Carried out molecular genetic experiments

DB: Guidance through the experiments; participated in writing the manuscript

RG: Design and coordination of the experiments; writing the manuscript

All authors read and approved the final manuscript.
